# Continuous EEG monitoring in ICU

**DOI:** 10.1186/s40560-018-0310-z

**Published:** 2018-07-17

**Authors:** Yuichi Kubota, Hidetoshi Nakamoto, Satoshi Egawa, Takakazu Kawamata

**Affiliations:** 1 Stroke and Epilepsy Center, TMG Asaka Medical Center, 1-1340 Mizonuma, Asaka, Saitama 351-8551 Japan; 2 Neurocritical Care Unit, TMG Asaka Medical Center, 1-1340 Mizonuma, Asaka, Saitama 351-8551 Japan; 3 Department of Neurosurgery, Tokyo Women’s Medical University, 8-1 Kawada cho, Shinjiku Tokyo, 162-8666 Japan

**Keywords:** Continuous EEG, Nonconvulsive status epilepticus (NCSE), Periodic discharges (PDs)

## Abstract

**Background:**

Continuous electroencephalogram (CEEG) monitoring is increasingly being used for brain monitoring in neurocritical care setting. This is because of the proven effectiveness of CEEG in diagnosing nonconvulsive status epilepticus (NCSE) as a cause of unexplained consciousness disorder. CEEG has been demonstrated to be effective in determining the response to, and outcome of, NCSE treatment.

**Main body:**

In this review article, the authors described the indication and methods of CEEG and diagnosis based on EEG pattern. As a condition characterized by unexplained consciousness disorder, NCSE is frequently encountered in the neurocritical care setting and is only accompanied by an altered EEG change without any clinically apparent manifestation, such as convulsion. Thus, it is considered a form of status epilepticus manifesting mainly with consciousness disorder. This is a diagnostic challenge but should not be overlooked as NCSE is a curable condition. However, CEEG is required for the correct diagnosis of NCSE, which is difficult to perform in daily clinical practice. There also are several challenges regarding urgent EEG monitoring in the intensive care unit setting, including system-related problems, such as the preparation of mobile EEG devices and collodion-applied electrodes; human resource-related problems, such as staffing of EEG technicians and physicians who can respond flexibly to unscheduled needs; and EEG-specific difficulties in interpretation/diagnosis. These issues preclude the wide spread of CEEG in daily practice.

**Conclusion:**

Recently, importance of CEEG was well accepted; however, no definitive diagnostic criteria exist for identifying EEG patterns suggestive of NCSE, especially the ambiguous significance of periodic discharges (PDs) further complicates the diagnosis of NCSE. Thus, analyzing the change in EEG patterns over time is important for the correct diagnosis of NCSE. Further studies are needed to collect sufficient CEEG data and assess the outcome of patients who have undergone therapeutic interventions.

## Background

### History: advent of digital EEG systems and EEG monitoring in the ICU

**Fig. 1:**
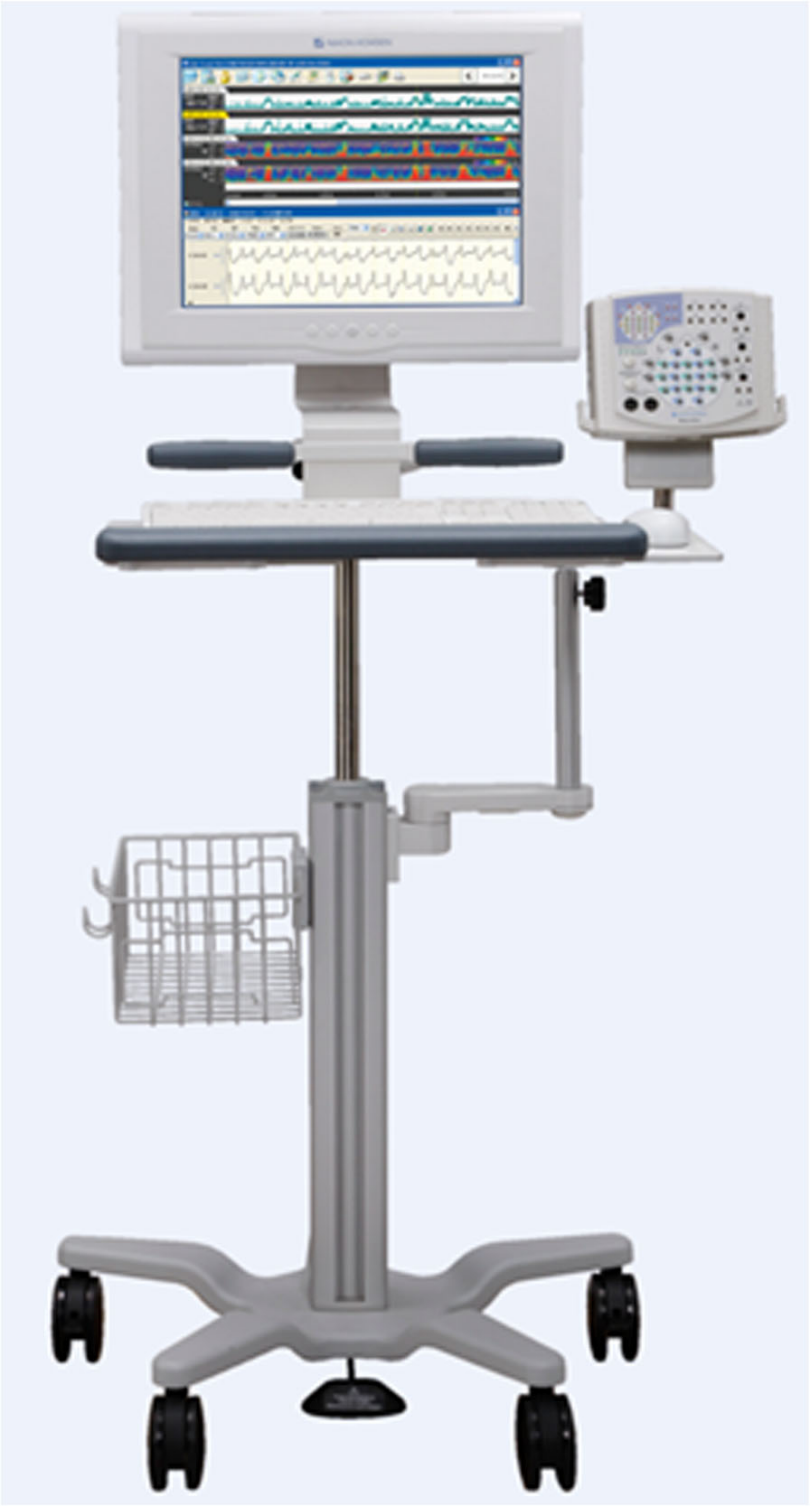
Mobile EEG system

For patients with unexplained consciousness disorder admitted to the intensive care unit (ICU), blood tests, blood gas analysis, and head computed tomography (CT) or magnetic resonance imaging (MRI) are generally performed but only provide findings at the point of examination. The brain undergoes continuous and dynamic changes, and therefore, continuous electroencephalogram (CEEG) monitoring is important as a method for assessing consciousness. Monitoring of EEG is a noninvasive procedure performed using electrodes attached to the scalp surface. Since 1990, the spread of digital EEG systems has enabled the filtering/refiltering of EEG waveforms, which has allowed EEG waveforms to be presented in a more easily readable format. Moreover, the advent of quantitative EEG displays, such as density spectral array (DSA) and compressed spectral array (CSA), has allowed us to readily detect seizure by color, instead of waveforms, from long-term EEG data. The subsequent advent of portable digital video EEG systems has enabled easy EEG measurement at any place, including emergency rooms and ICUs (Fig. 1). The recent expansion of hard disk capacity and network servers has also enabled the storage of massive data, such as long-term EEG data and concurrently recorded videos. These developments have led to the increased use of EEG in the ICU setting.

### The significance of CEEG in the ICU setting

The significance of CEEG measurement in the ICU setting includes (1) detection of nonconvulsive status epilepticus (NCSE) in patients with unexplained consciousness disorder or mental deterioration, (2) assessment of sedative/anesthetic state, (3) early detection of delayed cerebral ischemia associated with subarachnoid hemorrhage, and (4) assessment of the outcome of patients with postresuscitation encephalopathy or subsequent severe neurological disorders [1]. Especially useful for patients with NCSE, CEEG can detect changes in EEG over time, thereby enabling the early initiation of treatment, and can evaluate response to treatment, if administered, over time.

### CEEG procedures in the ICU setting—from patient selection to electrode attachment and monitoring

Patient selection is an important factor to be considered when performing CEEG. Performing the procedure on all patients with consciousness disorder will significantly increase the technical burden. Since the numbers of EEG machines and technicians available are always limited, it is necessary to select patients who require CEEG. The standard portable EEG is sufficient for consciousness disorders of known cause (e.g., irreversible stroke due to brainstem hemorrhage or other causes, metabolic disorders such as hypoglycemia, and drug intoxication). A questionnaire-based survey of American neurologists engaged in CEEG [2] showed that the procedure was most commonly used for patients with mental deterioration or coma after recent seizure (89%), subtle eye movement (85%), and mental deterioration or coma without seizure (68%).

**Fig. 2:**
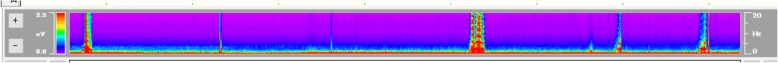
Example of density spectral array (DSA). The horizontal axis shows time, the vertical axis shows the frequency (Hz) of EEG, and color spectrum shows EEG amplitude. It changes from blue to red when EEG amplitude increases. Changes in color are associated with seizures or some artifacts

Digital EEG systems are required for CEEG monitoring. Current EEG systems have optional quantitative display functions, such as amplitude-integrated EEG (aEEG) and DSA, which allow detection of long-term changes in EEG signals at a glance and thus are useful for screening purposes (Fig. 2). An attached camera also allows simultaneous EEG measurement and video recording, as well as detection of EEG noises caused by the patient’s body movement, aspiration, or other factors.

For long-term EEG measurement, collodion-applied electrodes are usually used, instead of dish-type electrodes. Collodion-applied electrodes are preferred in the ICU setting because of possible electrode displacement during body repositioning, rehabilitation training, or other interventions performed by nurses, or due to sweating of the patient. When attaching electrodes, the scalp surface is wiped well with alcohol-soaked cotton and the electrodes are placed on the surface, which are then covered by a 2 × 2-cm piece of gauze and fixed with collodion. After the collodion dries, adhesive paste for electrodes is applied. A total of 21 electrodes, including 18 right and left electrodes (9 each), including earlobe electrodes and 3 midline electrodes, are used in accordance with the international 10–20 system. However, electrode attachment according to the 10-20 system may not be feasible in a busy emergency setting. In that case, fewer electrodes may be used for EEG measurement. A study comparing the 10-20 system and the use of fewer electrodes showed that the rate of detecting seizure from EEG signals was 93, 68, and 40% with 7, 4, and 1 electrodes, respectively [3]. Thus, CEEG can still be performed with fewer electrodes as long as it is understood that the use of fewer electrodes is associated with a somewhat decreased diagnostic yield. Monitoring time is an important factor affecting the examination results. Claassen et al. have reported that longer measurement time is associated with higher detection rate for NCSE, with a rate of 56% with 1-h and ≥ 80% with 12-h measurement, suggesting the need for longer measurement time for patients strongly suspected of having NCSE [4]. A recent study has also demonstrated that when CEEG is not available, a 30-min EEG measurement in the ICU provides a substantial diagnostic yield and leads to the detection of EEG activities associated with most types of status epilepticus [5].

Data from EEG are usually interpreted visually; aEEG and DSA can also be used for the quick screening of long-term EEG data, but in case of any abnormal finding, the visual analysis of EEG data is required to determine whether it is an artifact or a seizure pattern. When using aEEG for interpreting EEG data, it should be noted that the patient’s body movement and other factors may cause seizure patterns, resulting in false positive diagnosis [1].

### Underlying conditions of NCSE in the ICU setting

Features of NCSE are observed in various critically ill patients admitted to the ICU; NCSE is one of the brain responses associated with severe pathological conditions and is not a cause per se. Most cases of NCSE are associated with acute brain disorders, such as stroke, head trauma, and CNS infection, while some cases also occur after neurosurgical craniotomy [6]. On the other hand, a study in the surgical ICU setting showed that CEEG detected NCSE-related EEG patterns in 16% of the patients with consciousness disorder without brain abnormalities. Underlying disorders included failure of various organs, transplantation, and sepsis [7]. Further, NCSE associated with antibiotics such as cefepime, levofloxacin, and clarithromycin has also been reported [8–10].

#### Stroke

Stroke in itself seems to be associated with the risk of NCSE. Concerning ischemic stroke, all types of ischemia, that is, not only cortical ischemia but also lacunar infarction, have the possibility of developing subsequent NCSE. Among elderly critically ill patients, Litt et al. reported that 24 NCSE episodes were found, within them five patients only had lacunar infarction. However, the pathophysiology had not mentioned [11]. Furthermore, subarachnoid hemorrhage is a known cause of NCSE. The incidence of developing NCSE in subarachnoid hemorrhage patients is reported to range between 3 and 31% [12, 13]. Of note, the presence of periodic discharges or NCSE, as well as the absence of normal sleep architecture and reactivity, have been independently associated with poor neurological outcomes, defined as a modified Rankin Scale score greater than 4 [14]. Intracranial hemorrhage (ICH) patients occasionally develop seizures. Compared with deep ICH, lobar ICH including insular patients tends to develop NCSE more frequently. And also, intervention of craniotomy also developed NCSE [15].

#### Traumatic brain injury

Traumatic brain injury (TBI) is also associated with a risk of subsequent NCSE, which is being increasingly recognized as harmful. In a retrospective study of TBI patients undergoing CEEG, Claassen et al. found that 18% of patients experienced a seizure during the CEEG monitoring, all of which were subclinical seizures, while 8% developed NCSE [4]. On the other hand, in a pediatric population, Arndt et al. reported the usefulness of CEEG for the detection of subclinical early posttraumatic seizures; they found that subclinical seizures occurred in 16.1% of patients [16].

### Diagnosis of NCSE by CEEG

#### EEG terminology

**Fig. 3:**
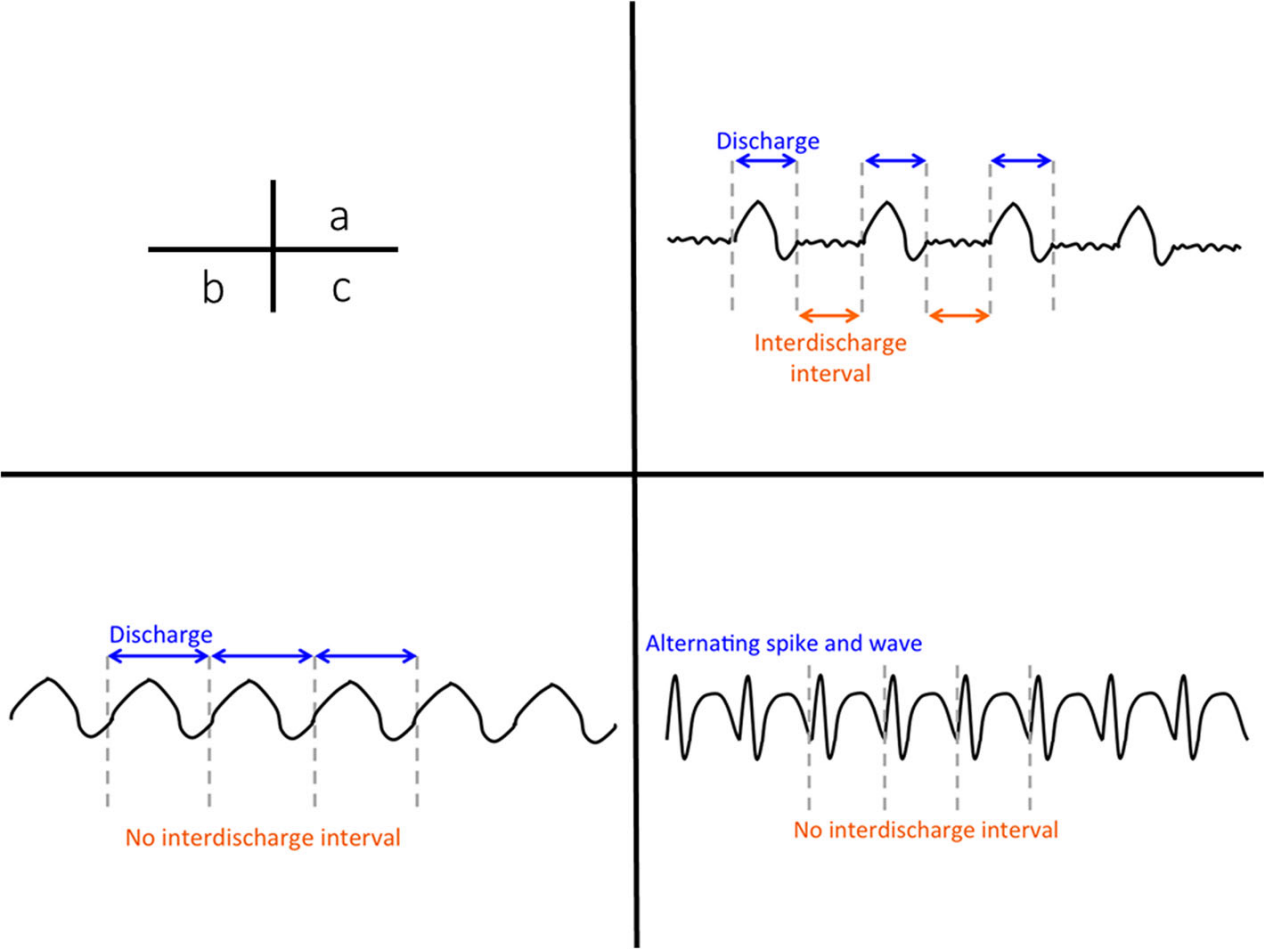
EEG pattern associated with NCSE. a: periodic discharges. b: rhythmic delta activity. c: spike-and-wave

Since NCSE does not manifest with overt convulsion, EEG interpretation plays an important role in its diagnosis. Moreover, long-term EEG monitoring shows that the EEG patterns of patients with consciousness disorder substantially fluctuate both temporally and spatially. It is also often difficult to determine whether an abnormal EEG pattern occurred during seizure, between seizures, or after seizure. With no established definition/classification of neurocritical EEG, such decisions are often based on the Standardized Critical Care EEG Terminology (2012), proposed by the American Clinical Neurophysiology Society [17]. This classification system simply categorizes EEG patterns observed in the neuro-ICU setting mainly by waveform and localization. It also avoids the use of clinical expressions, such as “during seizure,” “between seizures,” “epileptic,” and “triphasic wave,” and classifies EEG patterns based on waveforms. In the main term 1, EEG patterns are classified according to their localization into generalized, lateralized, bilateral independent, and multifocal patterns. Then, in the main term 2, patterns are classified according to their waveform morphology into periodic discharges (PDs), rhythmic delta activity (RDA), and spike-and-wave or sharp-and-wave (SW) (Fig. 3). The PD pattern is defined as the repeated occurrence of the same paroxysmal discharge at a relatively constant interval. The RDA pattern is defined as the persistence of a high-amplitude waveform of ≤ 4 Hz, without interval between discharges. The SW pattern is defined as the persistence of spike/sharp waves followed by slow waves. In addition to these classifications, sub-classifications are defined using modifiers, such as frequency, amplitude, continuity, interval, and polarity. After the main term classification, epileptic discharges and basic activities are classified.

This classification system aims to avoid biased EEG interpretation based only on clinical information, such as triphasic waves associated with hepatic encephalopathy, classifying EEG patterns based only on waveforms, as much as possible. The periodic lateralized epileptiform discharges (PLEDs) and generalized lateralized epileptiform discharges (GPEDs)/periodic patterns, according to the conventional classification system, are described as the lateralized periodic discharges (LPDs) and generalized periodic discharges (GPDs) in the current classification system, respectively.

#### Diagnosis

This EEG terminology does not mention which of the subsequent EEG patterns should be recognized as a NCSE pattern. The PD pattern was initially considered to reflect disrupted cortico-subcortical communication, mainly due to white matter lesions [18]. However, recent evidence suggests that the pattern can reflect both irreversible and recovering states [19].

#### Criteria for nonconvulsive seizure proposed by Chong (Fig. 4)

Among various recent studies on the diagnosis of NCSE based on EEG patterns, Chong et al. have defined three primary criteria for diagnosing NCSE, where NCSE is diagnosed by the persistence of these patterns for at least 10 s [20].

**Fig. 4:**
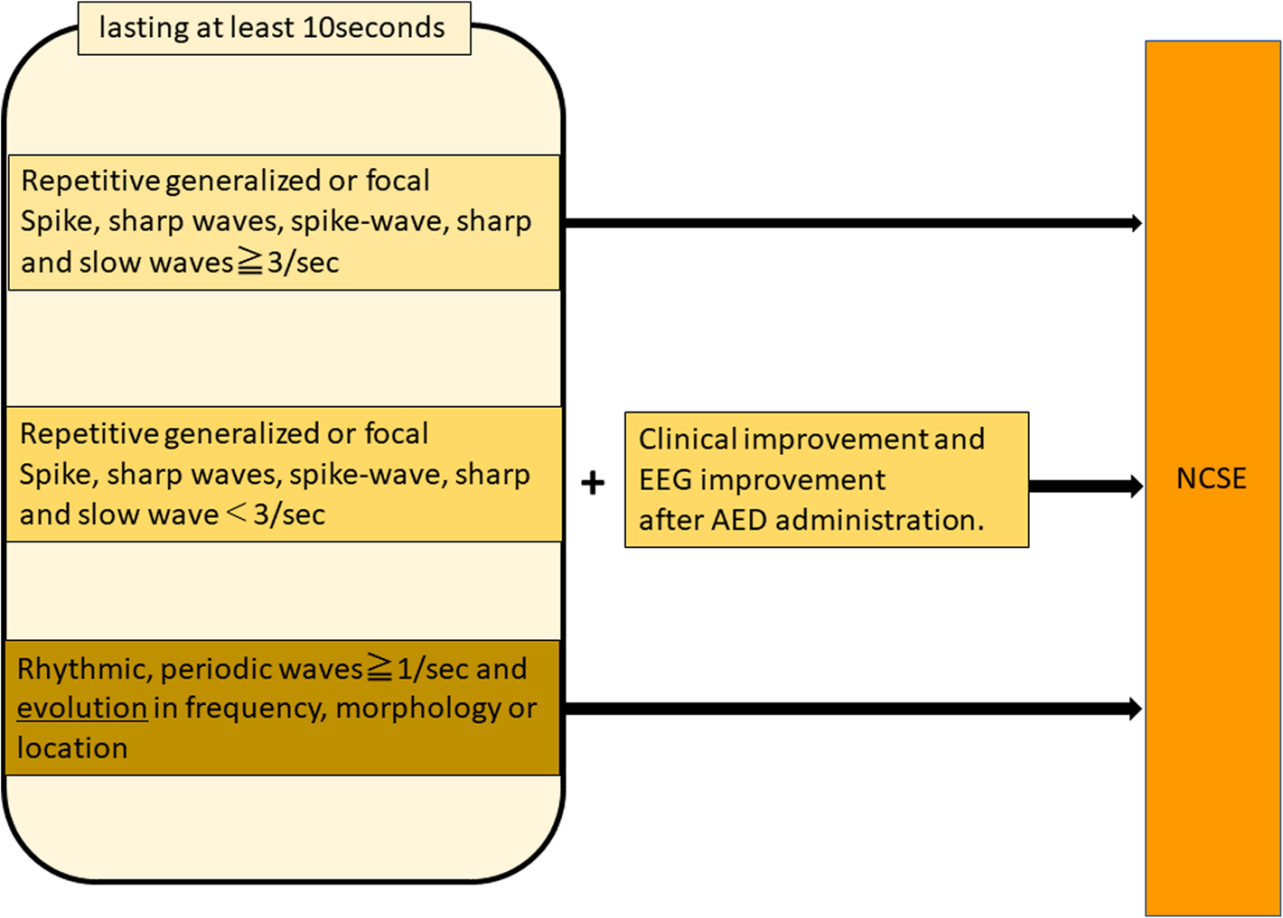
Chong’ criteria for NCSE

#### Sutter’s proposed NCSE criteria (Fig. 5)

According to Sutter et al., NCSE should ideally be diagnosed based on clinical symptoms and EEG findings, and the six criteria are required for diagnosing NCSE in adults [21].

**Fig. 5:**
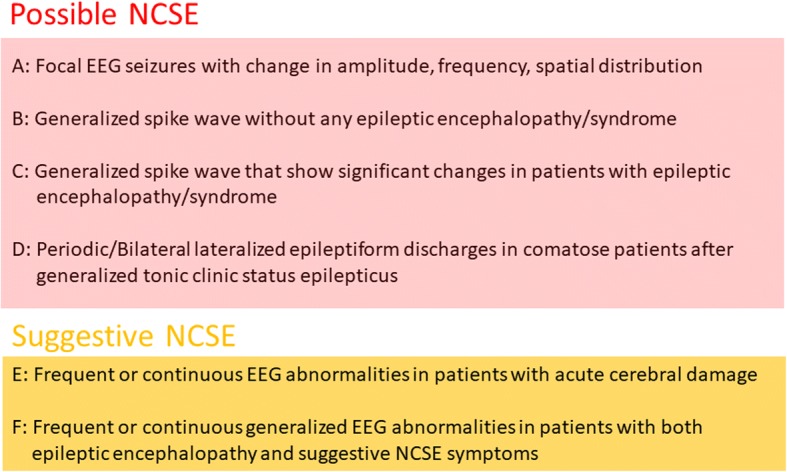
Sutter’s criteria for NCSE

#### Modified Salzburg EEG criteria for the diagnosis for NCSE (Fig. 6)

Among the various diagnostic criteria, the modified Salzburg Consensus Criteria for Non-Convulsive Status Epilepticus was proposed in 2015. Based on these criteria, NCSE is diagnosed by the occurrence of 25 PDs (2.5 Hz) per 10 s, spatio-temporal changes, and PDs and RDA associated with minor clinical symptoms. These are currently the most commonly referred diagnostic criteria [22].

**Fig. 6:**
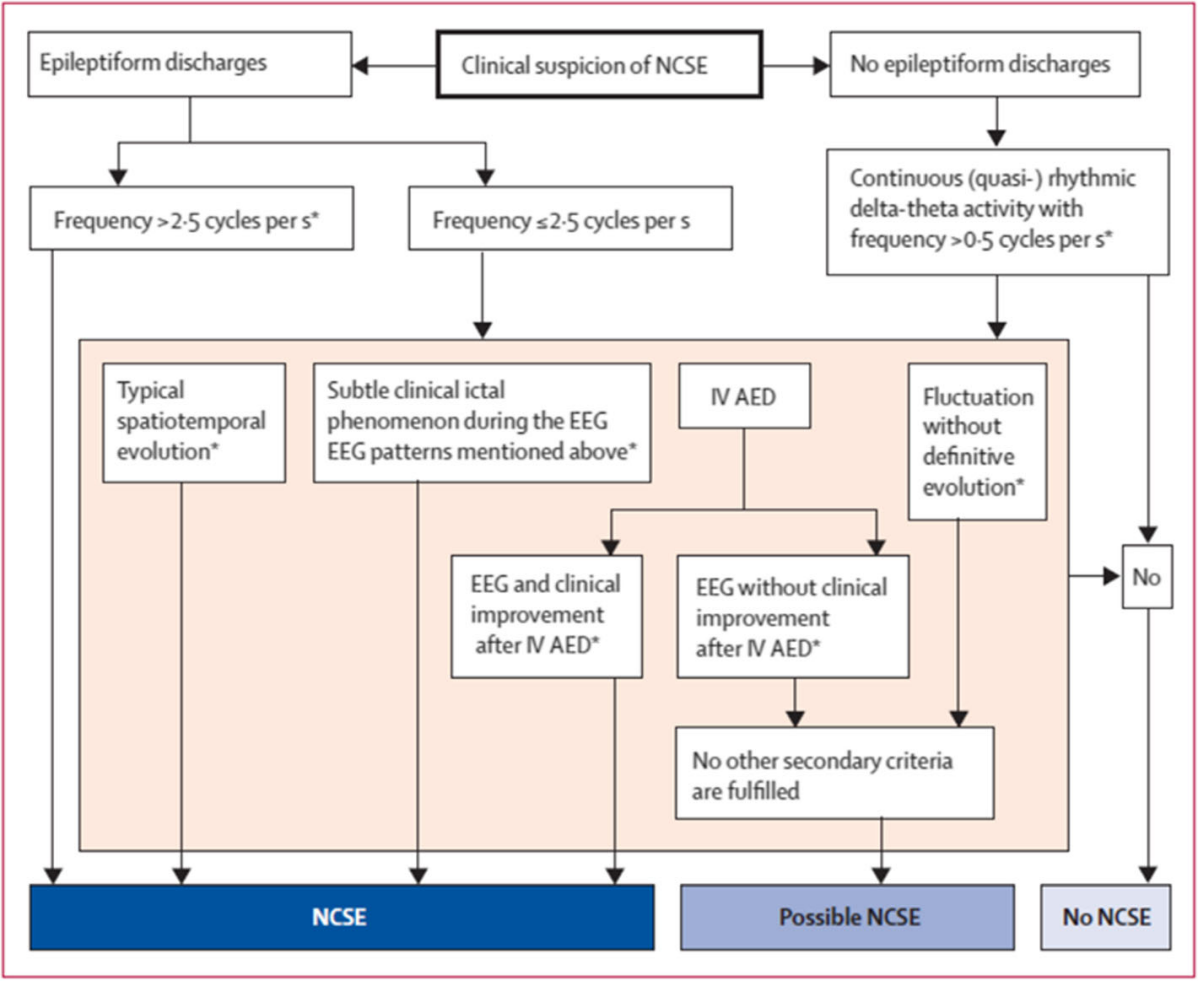
Modified Salzburg consensus for NCSE (from Leitinger et al., Lancet Neurol 2016;15:1054–62)

The authors had introduced CEEG in our hospital in 2013, which was its first introduction in Japan. The EEG terminology is based on the Standardized Critical Care EEG Terminology (2012), including PD (L or G), RDA (L or G), SW, and Evolution, which is defined as changes in the frequency of appearance of periodic/rhythmic patterns, many of which are increased in frequency and widened spatially. The diagnosis of NCSE is based on the aforementioned modified Salzburg Consensus Criteria for Non-Convulsive Status Epilepticus. The PD pattern should be interpreted with care as it is a “yellow flag” that can be observed during as well as between NCSE episodes. Changes in the number of cycles with PDs and altered spatial spread in EEG electrodes can lead to the diagnosis of NCSE and justify therapeutic intervention. In contrast, the LPD pattern (LPD static), conventionally referred to as the PLEDs proper, characterized by a constant frequency of PDs, is usually considered to represent an interval between seizures, and does not lead to active interventions, but may justify therapeutic intervention in cases of suspected NCSE based on clinical course and symptoms. In such cases, we believe that CEEG should be performed to detect any change in EEG waveforms. The “PLEDs plus” pattern, defined as rhythmic PDs, has been associated with seizure [12] and may prompt treatment for status epilepticus. When a therapeutic intervention does not lead to altered EEG patterns or improved clinical symptoms, it should be discontinued immediately in view of the possibility that the treatment itself has an adverse effect on the patient. In contrast, the RDA pattern represents intervals between NCSE episodes or recovery from seizure, and thus, only requires follow-up without active therapeutic intervention. However, the RDA or slow-wave pattern containing spike/sharp waves requires close follow-up as they may undergo subsequent changes or even progress to NCSE.

The EEG waveforms are constantly changing. Chong et al. have proposed the importance of the interictal-ictal continuum for the diagnosis of NCSE [20]. They mentioned that NCSE represents the continuum in which seizure patterns on EEG, such as Evolution and SW, are followed by patterns appearing between seizures, such as the periodic pattern and rhythmic activity, and vice versa. In this continuum, EEG patterns also undergo dynamic changes, reflecting repeated secondary nerve injuries. Therefore, the correct interpretation of EEG findings is very important to enable early therapeutic interventions and thereby prevent nerve injury at as early a stage as possible.

Consequent improvements in consciousness disorder and/or mental deterioration do not necessarily occur in parallel with improved EEG findings. While abnormal EEG findings that can be improved by antiepileptics are most likely to indicate NCSE, those not responsive to interventions suggest the presence of brain injury associated with other underlying conditions, such as post-cardiac arrest encephalopathy and severe head injury, and do not justify further active treatment. Thus, avoiding overdiagnosis of NCSE based only on EEG findings and consequent overtreatment with multiple antiepileptics is as important as actively diagnosing and treating NCSE.

### Our experience with CEEG in the ICU setting—the outcome of 70 consecutive cases of CEEG monitoring

Since 2013, we have performed 12-h or longer CEEG procedures on patients with unexplained consciousness disorder admitted to the ICU. Summarized below is the outcome of 70 consecutive cases of CEEG monitoring. The mean age of the patients was 64.4 years (range, 17–90 years). There were 38 patients with acute stroke, 19 with epilepsy (including post-stroke, post-brain tumor, and post-traumatic), 5 with acute head trauma, 2 with encephalitis, 2 with psychogenic seizure, and 4 with other conditions. Of all the patients, 32.85% (23 patients) were diagnosed with NCSE, based on EEG findings. The EEG findings included Evaluation in 8 patients, SPW in 1, LPDs (including PLEDs plus and increased LPD) in 13, and GPDs in 1.

Treatment for NCSE is based on the evaluation and management guidelines for status epilepticus proposed by the American Neurocritical Care Society [23]. The first-line treatment consists of fosphenytoin at a loading dose of 22.5 mg/kg or 15 mg phenytoin equivalent (PE)/kg, followed by checking EEG patterns for any improvement 12 h later, as well as measuring blood phenytoin concentration to check whether the concentration reaches the optimal level. Although the recommended phenytoin dose in the overseas literature is 20 mg PE/kg, we have used the 15 mg PE/kg dose and achieved a blood concentration of 10–15 μg/ml on the following day. In case of no improvement in the EEG findings or clinical symptoms, the second-line treatment with levetiracetam is added. Both phenytoin and fosphenytoin which is prodrug of phenytoin historically show the usefulness for status epilepticus [24, 25]. One recent study reported that status epilepticus was terminated with intravenous levetiracetam (LEV) in 68.75% of patients and with intravenous phenytoin in 83.3% of patients [26]. In current prospective study, levetiracetam also showed tolerability for status epilepticus without any major adverse effect [27]. In this report, 14 patients had convulsive SE (CSE), 11 had nonconvulsive SE (NCSE), and 5 had epilepsia partialis continua (EPC). The patients were given intravenous levetiracetam with dosages ranging between 1000 and 4000 mg/day. Twenty-nine of the patients continued to receive levetiracetam orally as maintenance treatment. Status epilepticus was terminated in 23 (76.6%) patients.

## Conclusion

While an increasing number of studies addresses classification of the EEG patterns observed in the neurocritical care setting, no standardized diagnostic criteria exist for identifying EEG patterns suggestive of NCSE. In particular, the ambiguous significance of PDs among EEG signals in the neurocritical care setting further complicates the diagnosis of NCSE. Although PDs are often associated with seizure, they can also be observed between seizures. Thus, analyzing the change in EEG patterns over time is important for the correct diagnosis of NCSE. Further studies are needed to collect sufficient CEEG data and assess the outcome of patients who have undergone therapeutic interventions.
